# Galectin-3 in Atrial Fibrillation: Mechanisms and Therapeutic Implications

**DOI:** 10.3390/ijms19040976

**Published:** 2018-03-25

**Authors:** Nicolas Clementy, Eric Piver, Arnaud Bisson, Clémentine Andre, Anne Bernard, Bertrand Pierre, Laurent Fauchier, Dominique Babuty

**Affiliations:** 1 Cardiology Department, Trousseau Hospital, François Rabelais University, 37000 Tours, France; arnaud.bisson37@gmail.com (A.B.); clementine2andre@gmail.com (C.A.); anne.bernard@univ-tours.fr (A.B.); b.pierre@chu-tours.fr (B.P.); laurent.fauchier@univ-tours.fr (L.F.); d.babuty@chu-tours.fr (D.B.); 2 Biochemistry Department, Trousseau Hospital, François Rabelais University, 37000 Tours, France; piver_e@univ-tours.fr

**Keywords:** galectin-3, atrial fibrillation, ablation

## Abstract

Maintenance of atrial fibrillation is a complex mechanism, including extensive electrical and structural remodeling of the atria which involves progressive fibrogenesis. Galectin-3 is a biomarker of fibrosis, and, thus, may be involved in atrial remodeling in atrial fibrillation patients. We review the role of galectin-3 in AF mechanisms and its potential therapeutic implications.

## 1. Introduction

Atrial fibrillation (AF), the most frequent of all arrhythmia is associated with structural, electrical, genomic, hormonal and autonomic atrial remodeling. Structural atrial remodeling includes hibernation phenomena, degenerative processes (aptotosis and fibrosis), and gap junction expression alteration [1]. More recently, the concept of fibrotic atrial cardiomyopathy has materialized, defined by a progressive invasion of atrial myocardium by fibrosis, which favors initiation and maintenance of AF [2]. Galectin-3 (Gal-3), a biomarker of fibrosis, has been shown to play a role in promoting fibrosis in patients with AF, and has emerged as a prognostic marker in this population. We review here the histologic and molecular mechanisms involved, and the subsequent issues in clinical practice.

## 2. Clinical Atrial Fibrillation

Atrial fibrillation, characterized by irregular, rapid and anarchical atrial electrical activity, either silent or responsible for symptoms (especially palpitations and/or dyspnea), is associated with a significant morbidity, an increased risk of thrombo-embolic events, especially strokes, hospitalizations for heart failure, and thus, an increased all-cause mortality [3]. Management of this disease has become a major issue in our healthcare systems.

Clinically, AF typically begins with paroxysmal episodes, which by arbitrary definition last less than seven days and may self-terminate spontaneously, or may need cardioversion, either with medications or an electrical shock [4]. It then may evolve towards so-called “persistent” AF, defined by more prolonged episodes, typically lasting more than 7 days. Finally, long-persistent AF is defined by a sustained arrhythmia lasting one year or more. The term of “permanent” AF is restrained to patients in whom normal sinus rhythm will not to be restored. Patients do evolve very differently. Some patients will present persistent AF as a first manifestation of the disease, while some will pass through several years of paroxysmal episodes, suggesting that the clinical history of the disease and its underlying pathophysiological mechanisms are not correlated. Although the mechanisms involved in the evolution of the disease remain unclear, AF has been shown to lead to electrical remodeling and progressive fibrosis of the atria, which in turn favor AF perpetuation and chronicization: “AF begets AF” [5,6].

## 3. Atrial Fibrillation Mechanisms

Different triggers can induce AF: abnormal automaticity, generally originated from pulmonary veins, induced by stretch or left atrium dilatation; functional localized reentry, usually induced by fibers anisotropy [7,8]. Perpetuation of AF may be related to rotors (localized reentries), multiple circuits of reentry, favored by atrial remodeling (multiple wavelet hypothesis), and electrophysiological remodeling secondary to autonomic nervous system, endocrinous and/or hemodynamic modifications [9]. Tissue heterogeneities, i.e., structural remodeling, are associated to slow conduction velocities and short refractory periods, i.e., electrical remodeling, the electrophysiological substrate for maintenance of arrhythmia [10,11,12,13].

### 3.1. Electrical Remodeling

At the cellular level, AF mechanisms are still controversial. They involve electrical remodeling of ion channels, gap junctions and abnormal Ca^2+^ handling from the sarcoplasmic reticulum. There is also evidence supporting the role of autonomic nervous system and inflammation [14]. Martins et al. have evaluated the rate of electrical and structural remodeling in an animal model of persistent AF. The increase of fibrillatory frequency varied between animals but its slope predicted the time at which AF becomes persistent, reflecting an abbreviation of action potential duration, changes in densities of ion currents, and a progressive increase in atrial dilatation, myocyte hypertrophy and atrial fibrosis [15].

Electrical remodeling involves major changes in action potentials: shortening of action potentials duration, acceleration of repolarization, but also a mismatching in the repolarization time course during rate changes [16]. Shortening of repolarization and refractory periods lead to a decrease of the wavelength, allowing formation of small circuits of reentry (micro-reentries). These changes alone may not explain the trigger of the arrhythmia. Gap junction cell coupling remodeling might be involved in the creation of the substrate needed to sustain reentry in single or multiple small circuits [17].

Autonomic remodeling plays a major role in electrical remodeling. The atria are innervated by parasympathetic and sympathetic systems: the extrinsic ganglia, the intrinsic ganglionated plexi network, and the baroreceptors. AF was found to lead to a heterogeneous increase in atrial sympathetic innervation [18]. Vagal activation is known to contribute to shortening of atrial refractoriness and subsequent AF [19]. Chen and colleagues have shown that the mechanisms by which autonomic changes promote AF include an enhanced automaticity, early/delayed after-depolarizations, and heterogenous shortening of refractory periods [20].

In an animal model study, high-resolution mapping demonstrated reduced left atrial AF electrical complexity along with reduced sympathetic activity after renal denervation [21]. Neuromodulation of autonomic tone is then to be investigated for treating AF.

### 3.2. Structural Remodeling

It is well known for several decades that AF is associated atrial cavity dilatation, atrial myocardium hypertrophy, and variable rarefaction of atrial cardiomyocytes, either focal or diffuse, replaced with fibrotic tissue. Whether structural remodeling precedes or follows the development of the arrhythmia is unclear. Interstitial fibrosis forces depolarization wave-front to slow down. The electrical impulse propagates through alternative pathways, in which cardiomyocytes have recovered excitability, inducing multiple reentrant circuits [1]. Atria are more prone to fibrogenesis than the ventricles. The relative abundance of collagen creates heterogeneous areas with different electrophysiological properties. Matrix metalloproteinases and pro-fibrotic signals stimulate the proliferation of fibroblasts and subsequent collagen synthesis and degradation. Mechanisms also involve altered expression of matrix proteins other than collagen (fibronectin 1, fibrillin 1), and deposition of proteoglycans and other extracellular matrix components.

At the stage of AF perpetuation (clinical persistent AF), left atrium is usually characterized by the presence of large areas of fibrosis. Magnetic resonance imaging (MRI), with the help of a specific dedicated software, remains the gold standard to identify atrial fibrotic tissue extend, but this technique lacks adapted availability in regular practice. The lack of feasibility due the rather scarce thickness of the atrial wall may also limit the analyses [22].

**Figure 1:**
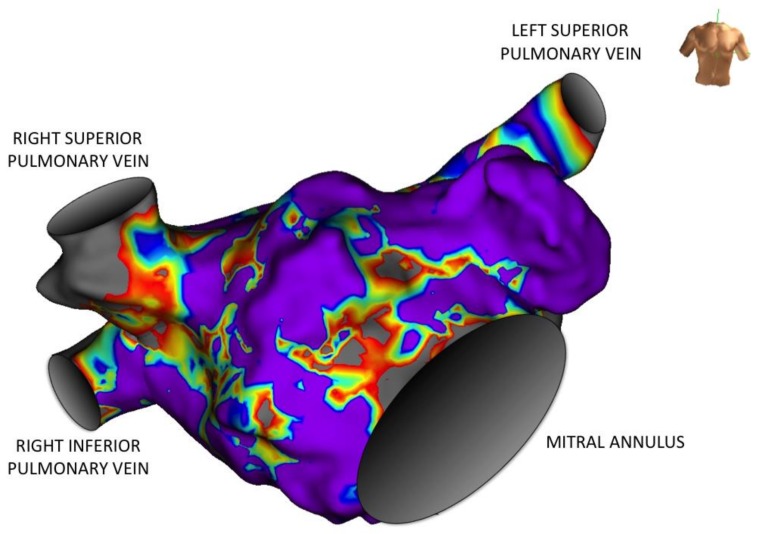
Three-dimensional voltage map of the left atrium obtained during invasive electrophysiological mapping (anterior view). The amplitude of local electrical activity is color-coded: from grey for low voltage areas to mauve for high voltage areas. Local amplitude correlates to the underlying atrial myocardial thickness, so that fibrotic areas translate into low voltage heterogeneous (“patchy”) anisotropic areas, here localized on the inter-atrial septum and the anterior wall.

Another quantitative method to assess atrial fibrosis is the invasive measurement of low voltage areas by electroanatomic mapping. Electroanatomic bipolar voltage mapping has been used as a surrogate to define scar [23,24,25]. A mapping diagnostic catheter is moved inside the atrial cavity, and dedicated software translates local mean signal amplitude into a color-coded image, i.e., a voltage map. Low voltage areas are then considered invaded by fibrosis (Figure 1). Extent of fibrosis is highly variable, and does not correlate with the clinical presentation of AF [26,27,28]. Haissaguerre et al. have shown that the fibrosis burden, particularly left atrial fibrosis, correlates independently with the number of electrophysiologically reentrant regions and the great majority of atrial fibrillation electrical drivers were found at the borders of fibrosis areas [29]. They have also shown a positive correlation with left atrial volume, and with uninterrupted AF duration. Sites with reentrant drivers showed a significantly higher disorganization index (i.e., a greater entropy), and a greater density of local fibrosis, showing that not all fibrosis carries the same proarrhythmic effect. Distinct tissue characteristics may form distinct electrical remodeling, favoring or not the creation of reentrant drivers.

Fibrosis tridimensional geometry may have an impact in AF perpetuation. Diffuse fibrosis has little effect on electrical wave fronts propagation. Conversely, “patchy” fibrosis can produce severe disruptions to propagating wave-fronts and rotor dynamics [30]. Propagation through highly fibrotic tissue is an example of percolation, the movement of a substance through a medium with random structure [31]. As demonstrated by Haissaguerre, “a wave-front passing through a medium with random fibrosis may then produce rate-dependent disruption of propagation and various reentries within a series of loosely coupled islands connected by strands” [29]. This phenomenon contributes to anisotropy. The cellular architecture of the myocardium is indeed anisotropic: myocardial cells are markedly elongated and form layers of tissue with sharply demarcated fiber orientation. Such fiber orientation induces a clear discrepancy between transverse and longitudinal electrical conduction velocities, translating into anisotropic properties in the propagation of the action potential and cells repolarization [32,33].

Cardiac magnetic resonance imaging lacking of availability and spatial resolution, and electro-anatomic mapping requiring an invasive procedure, simpler tools are needed to evaluate atrial structural remodeling in AF patients. Biomarkers seem to be promising in that setting.

## 4. Atrial Remodeling and Biomarkers

Atrial tissue is known to be more reactive to fibrosis than the ventricular one. Multiple interdependent signaling pathways lead to cardiac fibrosis: oxidative stress; inflammation via chemokines and cytokines; the renin-angiotensin-aldosterone system; transforming growth factor β (TGF-β) pathway; mechanical stretch via the expression of matrix metalloproteinases and their inhibitors [7]. Another signal pathway has been recently identified: epicardial adipocytes induce atrial fibrosis via adipo-fibrokines secretion [34].

In the biochemistry domain, fibrosis can be identified by evaluation of different biomarkers involved in extracellular matrix formation, such as collagen, fibronectin, laminin, fibrillin, or matrix metalloproteinases [35]. Among these markers, MMP-9, GDF-15, PIIINP and galectin-3 (Gal-3) appear as most promising [36].

Matrix metalloproteinase-9 (MMP-9) is an endopeptidase, synthesized and secreted in a monomeric form as zymogen. It can degrade the extracellular matrix components [37]. MMP-9 plasma levels are higher in AF patients and show a gradual increase from paroxysmal AF through persistent AF to permanent AF [38].

AF patients have also been shown to display higher values of GDF-15, a protein member of the transforming growth factor beta superfamily, than non-AF patients, GDF-15 being independently associated with paroxysmal AF [39].

Levels of PIIINP, the amino-terminal peptide of type III procollagen released into the blood during both synthesis and degradation of collagen type III, showed a nonlinear relationship with the risk of incident AF, both before and after risk adjustment [40].

Finally, studies on Gal-3 seem very encouraging regarding a clinical application in daily practice.

## 5. Galectin-3 Profibrotic Signaling

**Figure 2:**
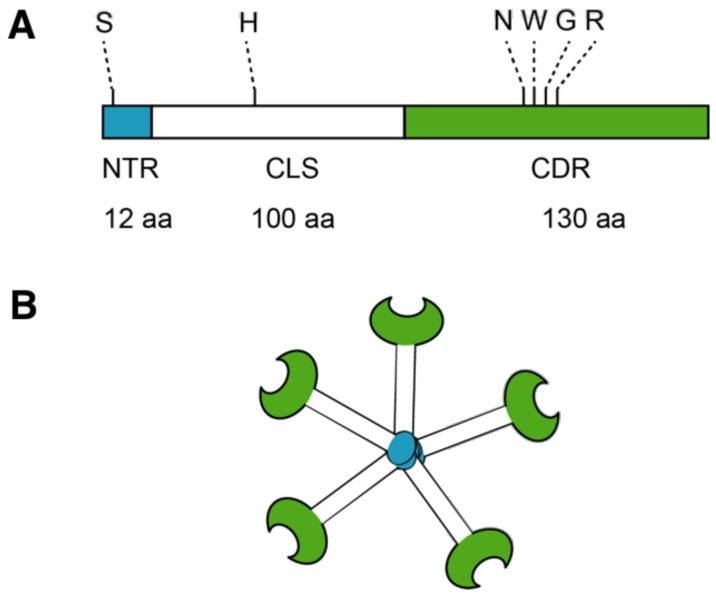
Structure of Galectin-3. (**A**) Galectin-3 protein structure consists of N terminal Domain (NTD), which has a N terminal Region of 12 amino acids (aa) and contains serine 6 (S) phosphorylation site. The carbohydrate recognition domain (CRD) 130 aa comprise the C-terminal and contains the NWGR motif; (**B**) Pentameric structure of Galectin-3.

Atrial fibrillation induces tissue injuries, which lead to increased Gal-3 synthesis and secretion. Gal-3 belongs to the galectins family, which are ß-galactoside-binding proteins, specifically binding to glycoproteins with *N*-acetyl-d-lactosamine disaccharide. In mammalians, 15 galectins were identified and named in accordance with their sequential discovery. They are classified in three groups according to the organization of their carbohydrate recognition domain (CRD). The first group of galectins (including Gal-1, -2, -5, -7, -10, -11, -13, -14, and -15) consists of homodimers through their single CRD, while in the second group galectins (Gal-4, -6, -8, -9, and -12) are structured in tandem-repeat with two distinct CRDs at their N- and C-termini. Gal-3, with a chimera structure, constitutes the third group. Gal-3 gene, named LGALS3, belongs to the chromosome 14 (locus q21-22). The *LGALS3* gene is composed of six exons and five introns spanning about 17 kilobases. Gal-3 molecule is a soluble protein of 29–35 kDa, and has a single CRD with a unique N-terminal domain (NTD) (Figure 2). The NTD consists of a relatively flexible structure of 7–14 repeating sequences and totals 110–130 amino acids (AA). It contains a N-terminal region (NTR) of 12 AA. In this NTR, the serine 6 can be phosphorylated by casein kinases 1 and 2, this regulation contributing to nuclear translocation and a reduction of affinity to its ligands [41]. Between the CRD and the NTD, a collagen-like sequence (CLS) consists of about 100 AA. This long tail contains the collagenase cleavable H-domain, in which histidine 64 is the site of action of matrix metalloproteinases (MMPs) such as MMP9 and MMP2 [42,43]. The CRD, consisting of 130 AA, forms a globular structure containing the binding site to carbohydrates, similar to other galectins. It also contains an Asp-Trp-Gly-Arg (NWGR) motif, similar to those described in anti-apoptotic BCL-2 proteins [44]. This sequence is also involved in the aggregation of Gal-3 molecules in the absence of ligand. Gal-3 affinity to its ligands is proportional to the number of lactosamine repeating units from the oligosaccharide structure.

Gal-3 was initially described as Mac-2 antigen in 1982 for its capacity to identify a macrophage sub-population. Actually, its identification in different pathway leads has given to Gal-3 a variety of names such as IgE binding protein, L-29, CBP30 or CBP35 [45]. It was eventually cloned in 1991 and subsequently recognized as a β-galactoside-binding lectin. Gal-3 has a pleiotropic distribution and could be found in the cytoplasm, in the nucleus and at the cell membrane, or as a pentameric circulating form [46]. The Gal-3 NTD drives self-oligomerization into a pentamer and may interact with extracellular and intracellular components. In the extracellular space, Gal-3 has the capacity to bind to different cells surface and extracellular matrix glycans in order to induce cell adhesion, migration, and growth regulation, mainly pro-apoptotic effects. In the intracellular space, Gal-3 regulates the cell cycle, inducing proliferation and anti-apoptotic effects. These combined actions, specific to Gal-3 in the galectin family, participate to a variety of pathophysiological processes involved in atrial fibrosis genesis: apoptosis, angiogenesis and inflammation [47]. Gal-3 is mainly produced by activated macrophages, mast cells, neutrophils and eosinophils [48]. In the heart, it is mainly expressed in fibroblasts.

**Figure 3:**
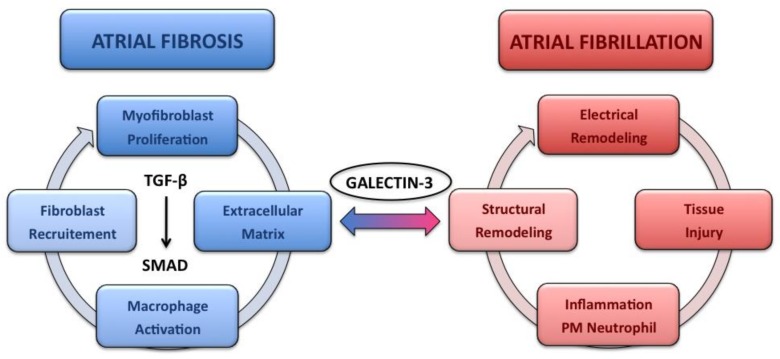
“Atrial fibrillation begets atrial fibrillation”. Galectin-3 production, promoted by atrial remodeling in atrial fibrillation patients, induces extracellular matrix production, mainly through the TGF-β/SMAD signaling pathway. Atrial fibrosis in return is associated with atrial dilatation, tissue anisotropy with heterogeneous electrical properties, which in turn favor atrial fibrillation initiation and maintenance.

The respective mechanisms by which Gal-3 exerts fibrogenic activity (fibroblast proliferation and collagen deposition) are not completely depicted. Extracellular pentameric Gal-3 interaction with profibrotic effectors such as TGF-β/SMAD could be a part of the pathway that initiates fibrogenesis. One of the hypotheses for activation of this pathway is the capacity of Gal-3 to form lectin-saccharide lattices on cell surfaces. TGF-β receptor entrapment within the lattice may amplify profibrotic signaling [49]. The TGF-β/SMAD pathway is well known to induce the recruitment, the activation and the transition of macrophages and mast cells, and the production of extracellular matrix by tubular epithelial cells, endothelial cells, mesengial cells, podocytes, fibroblasts and myofibroblasts [50] (Figure 3).

TGF-β is consistently activated in cardiac fibrosis [51]. TGF-β, a pleiotropic cytokine, exerts its effects by binding to two distinct receptors (TβRI and TβRII) with intrinsic serine/threonine kinase activity. It can be found in three isoforms (TGF-β1, 2 and 3) encoded by three distinct genes [52]. These three isoforms signal through the same cell surface receptors and share common cellular targets. However, TGF-β1 remains the predominant form in the heart, so that the role of TGF-β in cardiac fibrosis is probably limited to the β1 isoform. TGF-β1 is present in the normal heart as a latent complex, unable to associate with its receptors. Following cardiac injury, a relatively small amount of latent TGF-β is activated, sufficient to induce a maximal cellular response [53]. Proteases, such as plasmin, MMP-2 and MMP-9, and the matricellular protein TSP-1, can activate TGF-β and thus play a major role in atrial structural remodeling. Reactive oxygen species can also trigger TGF-β activation.

TGF-β stimulation induces myofibroblast trans-differentiation and improves extracellular matrix synthesis [54]. TGF-β has also matrix-preserving effects. It can induce the expression of protease inhibitors: plasminogen activator inhibitor (PAI-1), tissue inhibitors of metalloproteinases (TIMPs) [52]. Activated TGF-β binds to the active kinase domain of TβRII. It induced subsequent activation of TβRI by transphosphorylation of serine and threonine residues in the GS box of its cytoplasmic domain. This phosphorylation induces binding to R-SMADs (receptor-regulated). Receptor-associated R-SMADs then heterodimerize with common mediator SMAD. They convey signals from membrane to nucleus, where they bind to SMAD binding element (SBE) sequences located in different promoter regions of various genes regulating cell growth. After ligand stimulation, inhibitory SMADs (I-SMADs) translocate from the nucleus to the cytoplasm and bind to TβRI; to deactivate R-SMADs, causing inhibition of TGF-β signaling [55].

The role of SMAD-independent pathways in fibrotic cardiac remodeling remains unclear. Protein kinase B (Akt) consists of three closely related isoforms (Akt1, Akt2, and Akt3). It modulates cell growth, proliferation, and survival. Stimulation of PI3-K (p85 catalytic and p110 regulatory subunits) activates Akt, which phosphorylates proteins involved in the control of cell cycle, stimulating cell growth. The activation of Akt by TGF-β can inhibit the glycogen synthase kinase GSK-3β by increasing its phosphorylation. Increased phosphorylation of GSK-3β is associated with increased α-SMA (alpha smooth muscle actin, ACTA2), an increased transformation of fibroblasts into myofibroblasts, and subsequent fibrosis [56]. TGF-β1 can also increase protein kinase C α (PKCα) activity. TGF-β1-induced increased expression of α-SMA has been shown to be PKCα activity dependent [57]. In addition, the increased fibroblast—myofibroblast transdifferentiation via the TGF-β1–PKCα pathway also leads to an increased expression of phosphodiesterase 1A (PDE1A). Pharmacological inhibition of PDE1A can reduce the TGF-β1-induced increased expression of α-SMA, whereas phorbol-12-myristate-13-acetate (a PKCα activator) increases it. Upregulation of PKCα expression by TGF-β1 is also inhibited by PDE1A inhibition. TGF-β1 can then increase α-SMA expression and subsequent myofibroblast formation via a PDE1A-PKCα-dependent mechanism [58]. Negative regulation of TGF-β signaling may be a major therapeutic target in order to limit the development of atrial fibrosis [59].

Gal-3 may regulate the effects of TGF-β1, and other cytokines, by promoting the retention of their receptors on the atrial myofibroblast surface membrane, leading to an increased transcription of profibrotic molecules via stimulation of phosphorylation and nuclear translocation of the SMAD complex. Gal-3 may also act as a promoter of nuclear translocation of β-catenin via an inhibition of GSK-3β phosphorylating activity [47].

Gal-3 is then a pivotal actor of cardiac fibrosis, highly expressed in fibrotic tissues, and upregulated in chronic inflammatory and fibrotic conditions in human [60].

Mammalian models of cardiac fibrosis demonstrate high level of Gal-3, and its inhibition may prevent cardiac fibrosis [61]. Commercially available Gal-3 inhibitors may be needed. GM-CT-01 is a galactomannan that binds to Gal-3 carbohydrate-binding domain [62,63]. Takemoto et al. have shown that GM-CT reduced both electrical and structural remodeling in an AF sheep model [64]. The authors suggested a role of TGF-β1/SMAD pathway inhibition, reducing activation of myofibroblasts and subsequent collagen deposition [65].

Gal-3 levels correlate with risk factors of cardiovascular disease associated to chronic inflammatory conditions such as hypertension, diabetes or obesity, and were shown elevated in heart failure [66]. Gal-3 appears now with the recommended characteristics of a biomarker that could be useful in the cardiovascular domain [67,68,69,70].

## 6. Disease Progression and Therapeutic Implications

Galectin-3 has been well studied in AF and associated with the disease progression. Ho et al. have shown in a large cohort that higher Gal-3 levels were associated with increased risk of developing AF in the general population, although not after accounting for other risk factors [71]. In that study, the mean value of Gal-3 in patients developing AF was 15 ng/mL. We found in a population of AF patients that Gal-3 levels ≥15 ng/mL were present in older patients, female, and those with hypertension or diabetes [72]. Moreover, Gal-3 levels were higher in patients with a persistent form of AF (non self-terminating AF), suggesting a role in the maintenance of this arrhythmia. Similar results were found following a myocardial infarction [73].

In the supplementary data of the DECAAF study, left atrial volume was similar whatever the extent of atrial fibrosis [22]. In our initial study, no correlation between LA volume measured by echocardiography was established with the level of Gal-3 [72]. However, Gal-3 levels were shown to independently correlate with the extent of left atrial fibrosis detected with MRI [74].

Gal-3 may then help guiding the medical therapy. Renin-angiotensin-aldosterone system inhibitors, known to limit AF progression, may be used in patients with higher Gal-3 serum levels [75]. Gal-3 may even participate in the future to AF therapy as a therapeutic target. Takemoto and colleagues have indeed shown that Gal-3 inhibition decreased AF inducibility, but also increased the probability of spontaneous conversion to sinus rhythm during persistent AF [64]. Gal-3 inhibition may then act on structural remodeling by decreasing atrial dilatation and atrial fibrogenesis, but also on electrical remodeling (action potential duration shortening and atrial dominant frequency increase).

Therapy in AF also includes anticoagulation, and disease progression may be associated with an increased risk of stroke. It was shown that increased plasma levels of galectin-3 were associated with occurrence of postoperative strokes among female patients who undergo carotid endarterectomy [76]. Higher Gal-3 levels were also found in patients with acute ischemic stroke in a case-control study, and independently associated with a more severe disease and a poorer outcome [77,78]. Specifically in AF patients, an ARISTOTLE sub-study showed that patients in higher quartiles of Gal-3 had a higher risk of ischemic stroke, although the association was not independent [79]. Higher Gal-3 levels are actually associated with diabetes, hypertension, or heart failure, and thus higher CHADSVASC scores and subsequent risk of stroke. Whether patients with higher Gal-3 levels would benefit more from anticoagulation remains speculative.

Finally, Gal-3 may predict the substrate that needs to be treated during radiofrequency ablation. Patients with paroxysmal AF but higher Gal-3 levels might require more extensive intervention in addition to the simple isolation of pulmonary veins [80].

## 7. Galectin-3 and Atrial Fibrillation Ablation

Ablation of atrial fibrillation (AF) can be recommended in symptomatic patients, after failure of initial medical therapy [4]. Ablation consists mainly in an electrical isolation of pulmonary veins, using either radiofrequency energy or cryotherapy. This procedure can be associated with a defragmentation, i.e., an ablation of the proarrhythmic substrate with homogenization of low voltage patchy areas and of slow conduction. Depending on the clinical presentation of the disease, and the procedure method and endpoint, outcomes differ, with a much higher rate of recurrence after ablation in patients with a persistent form of AF [81]. Wu et al. found in a small cohort of 50 patients with persistent AF that Gal-3 concentration was an independent predictor of AF recurrence after ablation [82]. We also identified Gal-3 and left atrial diameter as independent predictive factors of recurrence, both parameters being related to structural atrial remodeling [83]. Left atrial diameter was known to be a strong predictor of recurrences after AF ablation, probably due to a parallel progression of fibrosis and LA enlargement [84]. But we found a distinct role of these 2 parameters, confirming the results of the DECAAF study in which the extent of fibrosis was not correlated to left atrial size [22,85].

The DECAAF study emphasized the role of atrial fibrosis quantified by MRI in AF ablation patients. However, MRI appears cost ineffective when compared to a simple dosage of Gal-3 serum level. There is also no clear evidence that the presence of late gadolinium enhancement actually identifies histological fibrosis within left atrium. Gal-3 level is directly related to fibrotic tissue activity [86]. Finally, MRI is not feasible in all centers, as it necessitates specific dedicated software, and not feasible in all patients, especially patients with implanted cardiac devices such as pacemakers and defibrillators.

The clinical history of AF appears less important than the nature and the amount of the atrial arrhythmogenic substrate, which determine the results after AF ablation. Higher Gal-3 levels in AF patients are thought to be related to obesity, hypertension or diabetes (metabolic syndrome), conditions also associated with an increased incidence of AF [87]. These metabolic conditions actually favor fibrogenesis. Gal-3 level may reflect the importance of arrhythmogenic substrate, no matter what initially prompted it [88].

An extensive ablation may not always be required, or may even be deleterious, in the absence of a significant atrial remodeling [89]. Gal-3 level might help predefining the ablation strategy, either more conservative or more extensive, in advance of the potential intervention.

## 8. Limitations

Galectin-3, as a ubiquitous protein, is not a specific of cardiac fibrosis. It is elevated in several conditions such as liver cirrhosis, lung fibrosis or chronic inflammatory diseases. Moreover, it is not specific of atrial myocardium and is elevated in heart failure and cardiomyopathies with an underlying ventricular structural disease. This is particularly important as AF and heart failure may be inter-dependant, one condition leading to the other one and vice versa. Analyses of Gal-3 levels in AF patients must then always be cautious of any of these other conditions potentially associated with extra-atrial fibrotic invasion.

## 9. Conclusions

Galectin-3 plays a major role in the progression of atrial fibrosis, and has thus become an essential prognostic biomarker in AF patients. Elevated Gal-3 levels correlate with a more advanced form of the disease, associated severe comorbidities, less efficacy of treatment, and worse outcomes. Galectin-3 inhibition may be a therapeutic target for AF patients in the future.

## Abbreviations

AF	Atrial fibrillation
Gal-3	Galectin-3
MRI	Magnetic resonance imaging
